# Green hemostatic sponge-like scaffold composed of soy protein and chitin for the treatment of epistaxis

**DOI:** 10.1016/j.mtbio.2022.100273

**Published:** 2022-04-30

**Authors:** Jon Jimenez-Martin, Kevin Las Heras, Alaitz Etxabide, Jone Uranga, Koro de la Caba, Pedro Guerrero, Manoli Igartua, Edorta Santos-Vizcaino, Rosa Maria Hernandez

**Affiliations:** a NanoBioCel Research Group, Laboratory of Pharmaceutics, School of Pharmacy, University of the Basque Country (UPV/EHU), Paseo de La Universidad 7, 01006 Vitoria Gasteiz, Spain; b Biomedical Research Networking Centre in Bioengineering, Biomaterials and Nanomedicine (CIBER-BBN), Institute of Health Carlos III, Madrid, Spain; c Bioaraba, NanoBioCel Research Group, Vitoria Gasteiz, Spain; d BIOMAT Research Group, University of the Basque Country (UPV/EHU), Escuela de Ingeniería de Gipuzkoa, Plaza de Europa 1, 20018 Donostia-San Sebastián, Spain; e BCMaterials, Basque Center for Materials, Applications and Nanostructures, UPV/EHU Science Park, 48940, Leioa, Spain; f Proteinmat Materials SL, Avenida de Tolosa 72, 20018 Donostia-San Sebastian, Spain

**Keywords:** Epistaxis, Nasal pack, Hemostasis, Sustainability

## Abstract

Epistaxis is one of the most common otorhinolaryngology emergencies worldwide. Although there are currently several treatments available, they present several disadvantages. This, in addition to the increasing social need of being environmentally respectful, led us to investigate whether a sponge-like scaffold (SP–CH) produced from natural by-products of the food industry — soy protein and β-chitin — can be employed as a nasal pack for the treatment of epistaxis. To evaluate the potential of our material as a nasal pack, it was compared with two of the most commonly used nasal packs in the clinic: a basic gauze and the gold standard Merocel®. Our SP-CH presented great physicochemical and mechanical properties, lost weight in aqueous medium, and could even partially degrade when incubated in blood. It was shown to be both biocompatible and hemocompatible *in vitro*, clearing up any doubt about its safety. It showed increased blood clotting capacity *in vitro*, as well as increased capacity to bind both red blood cells and platelets, compared to the standard gauze and Merocel®. Finally, a rat-tail amputation model revealed that our SP-CH could even reduce bleeding time *in vivo*. This work, carried out from a circular economy approach, demonstrates that a green strategy can be followed to manufacture nasal packs using valorized by-products of the food industry, with equal or even better hemostatic properties than the gold standard in the clinic.

## Graphical abstract

**Figure:**
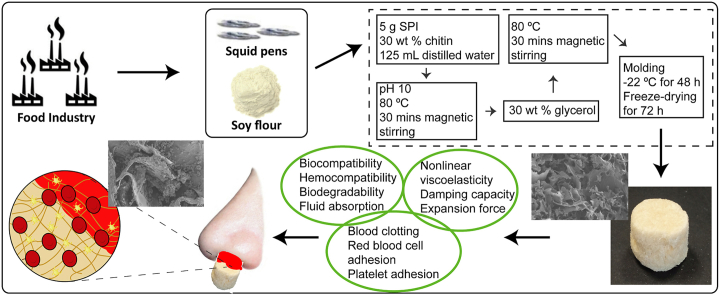

## 1 Introduction

Nasal hemorrhage, also known as epistaxis or nosebleed, is one of the most common otorhinolaryngology emergencies worldwide. It is estimated that 60% of the world's population will experience an epistaxis episode at least once in their lifetime, although only about 6%–10% of them would seek medical attention [1]. Despite being usually simple to treat, severe nosebleed might cause serious risks for the patient, especially for those over 70 years, for whom the incidence of these episodes is higher [2]. In addition to this, nasal injury is common after nasal surgery, for which treatment must be started quickly. Several methods are available to treat epistaxis, among which electrocautery, vasoconstrictors, surgical procedures and nasal packing arise as the most popular [3].

Nasal packing, which involves the insertion of a kind of tampon in the nasal cavity, is of special interest for the treatment of epistaxis, offering elevated rates of success [4]. Nevertheless, the choice of the most adequate nasal pack is vital for the outcome of the treatment. The ideal nasal pack should promote hemostasis while being comfortable for the patient, as well as hindering mucosal damage [5]. Therefore, alternatives to currently commercialized nasal packs are needed, since they are commonly nonabsorbable — causing patient's discomfort during removal — and act just by physical pressure tamponade, without any intrinsic hemostatic effect.

Additionally, sustainability is attracting more and more attention in all research areas — including health-related areas — with the aim of reducing the environmental load associated to processes and products. In this sense, environmental analysis is a useful tool to measure emissions throughout the product life. Its principal benefit is that it identifies the environmental impacts within the value chain, which could be redesigned to reduce the environmental burden. Most environmental assessments are studies in which emissions and resource use are classified into categories that can potentially harm the environment, such as global warming potential [6]. Numerous works have been carried out related to the processing of petrochemical-derived materials, but only a few related to those derived from biopolymers [[7], [8], [9], [10]].

In this context, natural polymers are arising as promising compounds for the development of hemostatic materials, being possible to manufacture them as scaffolds that mimic the extracellular matrix (ECM), which permits their reabsorption by the body [11]. These biopolymers are synthesized by living organisms such as plants, animals or microorganisms, and they generally offer good biocompatibility and biodegradability [12]. They might also possess different mechanisms to promote hemostasis.

Following this path, residual or unused biomass is an excellent source of proteins and polysaccharides. In this regard, soy protein (SP) is extracted from soybeans when soy oil is produced. In this process, soy flour is obtained as by-product, which is purified to obtain soy protein powder. Out of every 3 ​kg of soybean, 1 ​kg of soy protein is obtained [13]. In this study, soy protein was reinforced with chitin (CH), which provides higher stability and better mechanical properties. Chitin can be obtained from marine waste, such as shrimp and crab shells, following three processes [14]: deproteination in an alkaline medium to separate protein and polysaccharide, demineralization to eliminate inorganic matter using an acidic medium, and decoloration for pigment elimination. Interestingly, using squid pens as a source of chitin requires neither demineralization nor decoloration steps; thus, the extraction time, the employment of acid and basic solvents, and the voluminous wastewater discharge can be avoided, and the production costs, as well as the environmental load, can be considerably reduced.

In this regard, we recently developed and characterized a brand new sponge-like scaffold (SP–CH) based on a green strategy [15], using valorized by-products of the food industry. SPI is an abundant biopolymer that shows biocompatibility and low immunogenicity, as well as several peptides with interesting biological functions, such as the RGD-like sequence (Arg-Gly-Asp) containing peptides, providing great cell-adhesive properties [16]. For its part, CH — namely poly (β-(1–4)-N-acetyl-d-glucosamine) — also shows low toxicity due to its natural origin [17], and is biodegradable. These two biopolymers have been selected due to their broad valorization potential, since they are non-toxic, biocompatible, biodegradable, renewable, abundant and available at relatively low-cost, characteristics that provide them with a great value for the development of novel biosystems for health-related applications.

Although freeze-dried sponge-like structures with chitosan, crosslinked with glutaraldehyde, have shown blood-absorbing capacity suitable for haemostasis [18], in this work, chitin was used as reinforcement in order to provide soy protein with higher stability and better mechanical properties, avoiding the use of glutaraldehyde. Furthermore, since a brand new sponge-like scaffold (SP–CH) using valorized by-products of the food industry has been recently developed and characterized in a previous work [15], the aim here was to analyze its potential as a nasal pack for the treatment of epistaxis. The initial characterization of our SP-CH revealed its great physicochemical properties, with increased swelling capacity and partial degradability. Besides, the microstructure of the developed scaffolds could promote appropriate cell adhesion and proliferation, while remaining totally biocompatible. Even if several biopolymeric sponges has already been developed for the treatment of hemorrhagic wounds [[19], [20], [21]], hardly any of them seems to be directed towards the treatment of epistaxis [22,23], and none of them employs valorized products for its manufacturing.

Considering the excellent biomedical properties of the components of our SP-CH, we aimed to analyze its potential as a nasal pack for the treatment of epistaxis, comparing it with materials widely used as nasal packs during nosebleeds: a basic gauze and Merocel® (MRC), the nasal pack used worldwide as gold standard. We expect our material to present more adequate mechanical properties to be used as a nasal pack, in addition to a greater hemostatic capacity, compared to the aforementioned widely used products. To this end, we first compared their porosity, pore size, degradation, swelling profile and mechanical properties. Additionally, we performed *in vitro* cytotoxicity studies to confirm the safety of our SP-CH. Then, we investigated their hemostatic potential, including blood clotting capacity and the adhesion of erythrocytes and platelets to the materials. Finally, we evaluated the hemostatic efficacy of each material *in vivo*. On the other hand, in addition to the physicochemical and biological aspects, the environmental assessment was considered in this work as a key tool to evaluate the impacts of the scaffolds and the processes followed for their manufacture. One of the aims of using these materials was the valorization of side streams and bio-based waste into high value-added products, promoting resource efficiency, in line with the circular economy strategy, by creating new value chains. At the same time, the dependency on fossil-based polymers and GHG emissions are reduced in order to meet sustainable development goals. Therefore, this work was focused on researching unexploited opportunities to maximize the valorization potential of biological resources.

## 2 Materials and methods

### 2.1 Materials

Soy protein (SP), PROFAM 974, was supplied by ADM Protein Specialties Division (The Netherlands). The amino acid analysis as well as XPS and FTIR studies were performed in a previous work [15].

Chitin (CH) was extracted from fresh squid pens (Loligo sp.), which were treated with NaOH (1 ​M) at room temperature under continuous stirring for 24 ​h; afterwards, the solid fraction (CH) was washed with distilled water until neutral pH. Finally, CH was freeze-dried and milled to obtain the powder. Soy protein and chitin batches were those used and characterized in a previous work [14].

Glycerol, with a purity ≥99.5%, was supplied by Panreac (Barcelona, Spain). This polyol was employed as a plasticizer in order to disrupt intramolecular interactions among protein chains and facilitate intermolecular interactions between soy protein and chitin.

The properties of our SP-CH was compared with a standard gauze (Medicomp® 5 ​× ​5 ​cm, Hartmann bv, Nijmegen, The Netherlands) and with Merocel® (Medtronic Xomed, Jacksonville, Fla.). This commercial nasal pack, which is composed of hydroxylated polyvinyl acetate, and presented as a compressed and dehydrated sponge, is the primary nonabsorbable nasal pack in several emergency departments around the world [[24], [25], [26], [27]].

### 2.2 Preparation of the sponge-like scaffold (SP–CH)

In order to prepare the SP-CH, 5 ​g of SPI were initially mixed with 30 ​wt % CH (based on the SPI dry basis), followed by the addition of 125 ​mL of distilled water to the mixture. Next, NaOH (1 ​M) was employed to adjust the pH to 10, and the solution was heated at 80 ​°C for 30 ​min under magnetic stirring. Subsequently, 30 ​wt % glycerol (based on the SPI dry basis) was added to the blend. So as to achieve a homogeneous blend, it was maintained at 80 ​°C for other 30 ​min. The solution was then poured into moulds and maintained for 48 ​h in a freezer at −22 ​°C. Finally, it was freeze-dried for 72 ​h in order to complete the preparation of the SP-CH, which was eventually cut into discs using a hollow punch.

### 2.3 Environmental assessment

Environmental assessment was carried out according to ISO 14040 guidelines and recommendations: goal definition, inventory, impact assessment, and interpretation. The main goal of the study was to assess the environmental impact of the extraction of materials, the manufacturing of scaffolds and biodegradation as the end of life. The software used for this analysis was SimaPro 9.2.0.1 (PRé Consultants, The Netherlands). The materials used in the laboratory, the energy consumption during the extraction process, and the transportation of materials and residues were the resources and processes considered to develop the inventory phase. The environmental burden related to the production of the scaffolds was determined considering the energy (electricity) and materials (NaOH, glycerol, water) used in the extraction and manufacturing processes. Also transportation of materials, distilled water production and its transportation to the waste treatment plant after use were considered. Data were obtained from Ecoinvent v3 database, which provides data regarding energy production, transport and production of chemicals. The functional unit selected was 1 ​g. Based on the inventory data, environmental impacts were assessed according to the Hierarchist version of ReCiPe 2016, midpoint (H) v1.05. The impact categories under study were global warming, water consumption, land use, human carcinogenic toxicity, ozone formation (human health), terrestrial ecotoxicity, freshwater ecotoxicity, marine ecotoxicity, human non-carcinogenic toxicity, fine particulate matter formation, terrestrial acidification, freshwater eutrophication, marine eutrophication, mineral resource scarcity, fossil resource scarcity, ozone formation (terrestrial ecosystems), stratospheric ozone depletion, and ionizing radiation.

### 2.4 Blood obtention

Blood was acquired from healthy volunteers according to the protocols approved by the Ethics Committee for Researching Involving Biological Agents & GMOs (Procedure number: M30/2021/257) and the Ethics Committee for Research Involving Human Beings of the University of the Basque Country (Procedure number: M10/2021/256).

### 2.5 Pore size and porosity

A Hitachi S-4800 field emission scanning electron microscope (FE-SEM) (Hitachi High-Technologies Corporation, Tokyo, Japan) was employed to obtain the scanning electron microscopy (SEM) micrographs, using a beam accelerated voltage of 5 ​kV. All the tested materials were cut into discs of 8 ​mm diameter, mounted on a metal stub with adhesive tape and coated with gold under vacuum (JFC-1100) under an argon atmosphere before testing.

Image J software was used to measure pore diameters from SEM micrographs, in order to determine pore sizes. Between 75 and 250 randomly selected pores were measured for each material (n ​= ​3 samples per group), and the average pore size for each material was calculated.

To evaluate the porosity of the samples (15–20 ​mg, n ​= ​3 samples per group), a liquid displacement method was performed using 98% ethanol as the liquid medium, since it can diffuse through the samples without causing swelling or shrinkage [28]. V1 was defined as the initial volume of ethanol where the samples were submerged. Degassification was carried out for 5 ​min using a vacuum pump, so that the samples could be impregnated with the ethanol. V2 was defined as the total volume — ethanol plus soaked sample — after degasification. Last, ethanol-impregnated samples were removed and V3 was defined as the remaining volume of ethanol. The porosity (ε) of each sample was calculated using the equation below:

$$\varepsilon \left(\%\right)=\frac{V1-V3}{V2-V3}x\;100$$

### 2.6 Swelling capacity and liquid retention ratio under pressure (LRRP)

Swelling capacity of the materials (15–20 ​mg, n ​= ​4 samples per group) was calculated immersing preweighed samples (W_0_) of each material in 5 ​mL of PBS. The samples were weighed again (W_t_) after 5 ​s, 15 ​s, 30 ​s, 1 ​min, 5 ​min and 15 ​min, to obtain the swelling curves of each material. Swelling at each time point was calculated using the following equation:

$$Swelling\left(\%\right)=\frac{Wt-W0}{W0}x\;100$$

Liquid retention ratio under pressure (LRRP) was calculated in order to evaluate the ability of each material to retain the preabsorbed liquid when a certain pressure is applied. Samples of each material (20–25 ​mg, n ​= ​6 samples per group) were immersed in PBS to absorb as much liquid as possible, and then they were weighed (W_0_). Next, known weights — 10 ​g, 15 ​g, 30 ​g and 40 ​g — were serially placed upon the wet material, carefully collecting the released liquid and weighting the sample again. This procedure was repeated for each weight (W_X_). Then, LRRP was obtained using the following equation:

$$LRRP\left(\%\right)=\frac{Wx}{W0}x100$$

### 2.7 Weight loss in aqueous medium

Weight loss in aqueous medium was studied by immersing preweighed (W0) samples (20–25 ​mg, n ​= ​4 samples per group) of each material in 2 ​mL PBS, and culturing them at 37 ​°C for different periods of time — 2, 5, 9 and 14 days —. After removing the samples from PBS, they were first lyophilized and then weighed (Wf), in order to calculate the remaining weight (%). Weight loss in aqueous medium was expressed as the remaining weight of the incubated and lyophilized samples with respect to their initial weight, according to the following equation:

$$Remaining\;weight\left(\%\right)=\frac{Wf}{W0}x100$$

### 2.8 Mechanical characterization

The mechanical properties of the dry materials were tested by compression test and cyclic compression test (35–45 ​mg, n ​= ​5 samples per group). An Instron 5969 mechanical tester — equipped with a 50 ​N load cell — was used at a compressive rate of 0.5 ​mm ​s^−1^ up to 70% strain. As MRC is commercialized in lyophilized form, it was expanded before the test, by wetting and completely drying it off. Several parameters were calculated in order to compare the mechanical properties of the materials:

The maximum stress of each cycle relative to the maximum stress of the first cycle was calculated as follows:

$$Relative\;stress\left(\%\right)=\frac{Stress\left(cycle\;x\right)}{Stress\left(cycle\;1\right)}x100$$

Young's modulus was calculated for all the strains of the first cycle of each biomaterial, using the following equation for each point of the stress-strain curve:

$$Young^{\prime }s\;modulus\left(kPa\right)=\frac{Stress\left(kPa\right)}{\left[Strain\left(\%\right)/100\right]}$$

Damping coefficient was calculated by means of the stress-strain curves of the materials, both for cycle 1 and for cycle 10. The following equation was used:

$$Damping\;coefficient=\frac{D}{U}$$

where *D* is the dissipated energy (area between the loading and unloading curves) and *U* is the total input energy (area under the loading curve). All the areas under the curve where calculated using the software GraphPad Prism 8.0.1.

Besides, an expansion force test was performed, hydrating samples of dry materials (35–45 ​mg, n ​= ​5 samples per group) and recording the upwards stress displayed by the expansion of the material during water absorption.

### 2.9 Cytotoxicity studies

Cytotoxicity of the materials was assessed according to the ISO 10993-5:2009 guidelines for biological evaluation of medical devices. Both direct — direct contact with cells — and indirect — cells exposed to a conditioned medium of each material — cytotoxicity assays were performed. In both cases viability of L-929 fibroblasts (ATCC® 30–2003™) was measured through CCK-8 reagent (Sigma-Aldrich, Spain). After incubating the cells with CCK-8 solution in medium (1:11) for 4 ​h, the absorbance was read with a plate reader (Infinite® 200 PRO series, Tecan Trading AG, Männedorf, Switzerland) at 450 ​nm, using 650 ​nm as the reference wavelength. Cells that were not exposed to the materials — 100% viability — were used as a control group.

In the direct cytotoxicity assay, 35,000 ​cells were seeded per well in a 24-well plate, using 500 μL/well of EMEM complete medium. Next, the cells were incubated for 24 ​h at 37 ​°C. After removing the medium, 1 ​mL of fresh medium was added and each material (10–15 ​mg, n ​= ​4 samples per group) was hydrated and placed in a transwell to bring it into direct contact with the bottom of the well. The materials were removed after 48 ​h of incubation, replacing the medium with 300 μL/well of CCK-8 reagent.

In the case of the indirect cytotoxicity assay, L-929 fibroblasts were exposed to conditioned medium of each material (n ​= ​5 samples per group), obtained incubating 100 ​mg of each material with 500 ​μL of EMEM at 37 ​°C for 24 ​h. Firstly, a 96-well plate was used to seed 5000 ​cells/well in 100 μL/well of medium, and the plates were incubated for 24 ​h at 37 ​°C. After, the medium was replaced with 100 μL/well of the corresponding conditioned medium, and the plate was straightaway incubated at 37 ​°C for 24 ​h. Eventually, the conditioned medium was removed, and 110 μL/well of CCK-8 reagent was added.

### 2.10 Blood swelling and degradation in blood

Maximum blood swelling capacity was tested by immersing preweighed (W0) samples (15–20 ​mg, n ​= ​5 samples per group) of the materials in 500 ​μL of blood. After 2 ​min, to allow complete blood absorption, samples were weighed again (Wf). The maximum swelling capacity (%) was calculated using the following equation:

$$Swelling\left(\%\right)=\frac{Wf}{W0}x\;100$$

To evaluate the degradation of the materials in blood, preweighed (W0) samples (15–25 ​mg, n ​= ​4 samples per group) of each material were placed in 24-well plates, using one plate for each time-point tested — 2, 4 and 7 days —. Blood was added to each sample in an amount of a 120% of its maximum blood swelling capacity, and the plates where incubated at 37 ​°C. At each time-point, samples were washed out with PBS, lyophilized and weighed (Wf). Degradation in blood was expressed as the remaining weight of the incubated and lyophilized samples with respect to their initial weight, according to the following equation:

$$Remaining\;weight\left(\%\right)=\frac{Wf}{W0}x\;100$$

### 2.11 Whole blood clotting *in vitro*

Hemoglobin content of the samples was quantified using a Hemoglobin Assay Kit (Sigma-Aldrich, USA), by measuring absorbance of the samples (Is) with a plate reader (Infinite® 200 PRO series, Tecan Trading AG, Männedorf, Switzerland) at 400 ​nm.

For the whole blood clotting test, citrated whole blood — 9:1 whole blood to 3.8% sodium citrate — was obtained from a healthy human donor. First, 0.2 ​mL of the citrated whole blood was added to preheated (30 ​min at 37 ​°C) samples of the materials disposed in a 24 well plate (25–35 ​mg, n ​= ​5 samples per group). After this, 20 ​μL of CaCl_2_ (0.2 ​M) were added to start coagulation. The plate with the materials was incubated under 30 ​rpm agitation for 10 ​min at 37 ​°C. Afterwards, 2 ​mL of deionized water were added in each well to hemolyze the red blood cells (RBCs) that were not within the clot formed in the material. The deionized water containing the non-adhered and hemolyzed RBCs was collected and its hemoglobin content was quantified. 5 ​μL of CaCl_2_ (0.2 ​M) and 50 ​μL of citrated whole blood were added to 750 ​μL of deionized water, and the absorbance of this solution was used as the reference value (Ir). The absorbance of an empty well was also measured (Io). Blood Clotting Index (BCI) of each sample was calculated using the following equation:

$$BCI\;\left(\%\right)=\frac{\left(Is-Io\right)}{\left(Ir-Io\right)}x\;100$$

with the purpose of visually evaluating the clotting capacity of each material, 150 ​mg of each material were disposed in Eppendorf tubes, subsequently adding 500 ​μL of citrated whole blood and 500 ​μL of CaCl_2_ (10 ​mM). After incubating the Eppendorf tubes for 1 ​min at 37 ​°C, they were turned upside down and gently shaken to observe the formed clots.

### 2.12 Hemolysis assay *in vitro*

For the assessment of hemocompatibility *in vitro*, citrated whole blood — 9:1 whole blood to 3.8% sodium citrate — from a healthy donor was obtained and diluted 1:5 in normal saline. Samples of each material (10–15 ​mg, n ​= ​6 samples per group) were disposed in a 24-well plate. Diluted whole blood was added to each sample in an amount of the 80% of each material's swelling capacity. The plate was incubated for 1 ​h at 37 ​°C. Samples were moved to conical centrifuge tubes and centrifuged for 10 ​min at 3000 ​rpm. The supernatant of each sample was collected and its hemoglobin content was quantified as described in section 2.8. 150 ​μL of whole blood was added to deionized water and to normal saline, in order to use these solutions as positive (Ir) and negative (Io) controls, respectively. Hemolysis rate was calculated using the following equation:

$$Hemolysis\left(\%\right)=\frac{\left(Is-Io\right)}{\left(Ir-Io\right)}x\;100$$

### 2.13 Red blood cell adhesion *in vitro*

To evaluate RBC adhesion to the material, citrated whole blood — 9:1 whole blood to 3.8% sodium citrate — was collected from a healthy donor. Samples of each material were cut (15–20 ​mg, n ​= ​6 samples per group) and each sample was placed in a Petri dish. Citrated whole blood was added to each sample in an amount of the 80% of each material's swelling capacity, and they were incubated for 5 ​min at 37 ​°C. Next, 5–10 ​mL of deionized water were gently added by the edge of the dish until the water touched the sample and blood started to flow. More deionized water was added until a total volume of 50 ​mL for each sample. Each material was carefully moved to a clean Petri dish, and the liquid in each old Petri dish, — containing the RBCs that could not adhere to the sample —, was collected. The hemoglobin content of this solution was quantified as described in section 2.8. This measurement accounts for *hemoglobin outside* the material, that is, the RBCs that could not adhere to the material.

Afterwards, *hemoglobin within* the materials was measured, which accounts for the amount of RBCs adhered to each sample. 10 ​mL of deionized water were added straightly on the material — so that the previously adhered RBCs would be released — and the hemoglobin content in the resultant solution was quantified as described in section 2.8.

For both the *hemoglobin outside* and *within* the material, a positive control was prepared by mixing 200 ​μL of blood and 50 ​mL of deionized water. Hemoglobin concentration (mg dL^−1^) of each sample was calculated using the following equation provided by the Hemoglobin Assay Kit, and results of each sample were normalized against the blood volume added to the control group:

$$\left[Hemoglobin\right]=\frac{\left(Is-Io\right)}{\left(Ir-Io\right)}x\;100\frac{mg}{dL}x\;df$$

where *Is* is the absorbance of the tested solution; *Io* is the absorbance of the blank (water); *Ir* is the absorbance of the calibrator provided in the Hemoglobin Assay Kit; 100 ​mg/dL is the concentration of the diluted calibrator; *df* is the dilution factor, which was calculated for each sample depending on the volume of blood and deionized water added.

Citrated whole blood was also added to samples of each material (n ​= ​3 samples per group) and the adhesion of RBCs was visually evaluated using SEM micrographs, obtained as described in section 2.2.

### 2.14 Platelet adhesion *in vitro*

Adhesion of platelets to the materials was studied through a Lactate Dehydrogenase (LDH) Assay Kit (Sigma-Aldrich, USA), which allows to quantify platelet adhesion by measuring the LDH released by platelets when they are lysed, according to a reported method [29]. Briefly, citrated whole blood — 9:1 whole blood to 3.8% sodium citrate — was obtained from a healthy donor. After centrifuging it at 480 ​g for 10 ​min at 4 ​°C with 4 a/d, platelet-rich plasma (PRP) was obtained. The serum above the buffy coat was collected from the centrifuged blood samples and kept in citrated tubes. Samples of each material (15–20 ​mg, n ​= ​5 samples per group) were disposed in a 24-well plate and PRP was added to each sample in an amount of the 80% of each material's swelling capacity, and they were incubated for 30 ​min at 37 ​°C. Normal saline was gently added to each well until the samples were immersed, so that the non-adhered platelets could be removed from the material, and all samples were moved to a new 24-well plate. 1 ​mL of Triton X-100 1%-PBS was added straightly on the material, in order to lyse the platelets that had adhered to the material. After incubation for 1 ​h at 37 ​°C, each solution of Triton and lysed platelets was collected and LDH content was measured according to the protocol provided by the manufacturer (Sigma-Aldrich, USA) of the LDH Kit. To prepare the negative (Io) and positive (Ir) controls, 200 ​μL of PRP were added to 1 ​mL of PBS and Triton X-100 1%, respectively. Absorbances of the lysed platelets of each samples (Is) were measured with a plate reader (Infinite® 200 PRO series, Tecan Trading AG, Männedorf, Switzerland) at 400 ​nm, and normalized against the PRP volume added to the controls. LDH release (%) relative to the positive control was calculated using the following equation:

$$LDH\;release\left(\%\right)=\frac{\left(Is-Io\right)}{\left(Ir-Io\right)}x\;100$$

PRP was also added to samples of each material (n ​= ​3 samples per group) and the platelet adhesion was visually evaluated using SEM micrographs, obtained as described in section 2.4.

### 2.15 *IN VIVO* hemostatic efficacy

To evaluate the hemostatic efficacy *in vivo*, a rat-tail amputation model was used. This experiment was conducted following the protocols approved by the Institutional Ethical Committee for Animal Experimentation of the University of the Basque Country (Procedure number: M20/2021/362). Wistar rats — with weights between 250 and 300 ​g (Janvier Labs, Le Genest-Saint-Isle, France) — were anesthetized with isoflurane (Isoflo®, Esteve, Spain), and 2.5 ​cm from the end of their tail was cut using a scalpel. The tail was immediately placed in air for 10 ​s to guarantee normal blood loss, after which the wound was brought into direct contact with the corresponding preweighed material (250–300 ​mg, n ​= ​6 rats per group), holding it with a preweighed gauze (900–950 ​mg). The wound was uncovered every minute to check if bleeding persisted. For this reason, bleeding time (min) was assessed by assigning a score to each period at which bleeding ceased, as follows: 0–3 ​min: 1; 3–6 ​min: 2; 6–9 ​min: 3; >9 ​min: 4. Therefore, a lower bleeding time score represents a faster hemostatic effect. The blood-impregnated samples were weighed again to calculate total blood loss (g).

### 2.16 Statistical analysis

Mean ​± ​standard deviation was used to express the results. In the case of normally distributed data, results were analyzed through a one-way ANOVA test. Bonferroni or Tamhane post-hoc analysis was applied based on the Levene test for the homogeneity of variances. In reverse, non-normally distributed data was analyzed by Mann–Whitney's nonparametric analysis. Sample size of each experiment is indicated in the corresponding materials and methods section. p ​< ​0.05 was considered statistically significant. Moreover, all the statistical computations were performed using SPSS 25.0 (SPSS®, Inc., Chicago, IL, USA).

## 3 Results and discussion

### 3.1 Environmental assessment

The increasing environmental concern has led to assess the suitability of biodegradable polymers extracted from natural and renewable resources. Therefore, biopolymers derived from residual biomass have become an attractive alternative due to their abundance, cost and biodegradability. In this context, CH and soy protein were used in this work. On the one hand, CH can be obtained from shrimp, crabs or squid pens, among others. It should be noted that the CH content varies significantly depending on the source employed for its extraction. When CH is obtained from crustacean shells, CH content is around 20% after a demineralization process; however, the CH content from squid pens can reach 50%, with a mineral content lower than 3% [30]. On the other hand, soy protein, a by-product from soy oil production, can be purified to obtain SPI. In this process, soy flour is obtained as by-product, which is purified to obtain soy protein powder after centrifugation, acidification and neutralization processes. Although the properties of some products based on natural materials differ between batches, in the case of the extraction of chitin from *Loligo* sp., the traceability of this process has been confirmed. We follow a standardized process that allows us to obtain sponges without differences between batches, showing the same degree of acetylation and crystallinity [14]. To determine the environmental load associated with CH and soybean residue valorization, the environmental loads associated with those processes were considered.

All the abovementioned processes were considered to obtain the global environmental results of scaffolds production reported in Table 1. The results showed that terrestrial ecotoxicity, global warming and ionizing radiation caused low environmental load in the production of scaffolds, while the other categories contributed minimally to the overall environmental burden. We should note that the environmental burden of terrestrial ecotoxicity is related to soybean production and the generation of energy needed to obtain CH.

**Table 1:** Impact category values measured in the production of the scaffolds.

Impact Category	Unit	Total
Global warming	kg CO_2_ ​eq	1.7517401
Stratospheric ozone depletion	kg CFC11 eq	8.11E-07
Ionizing radiation	kBq Co-60 eq	0.99109783
Ozone formation, human health	kg NO_x_ ​eq	0.00560783
Fine particulate matter formation	kg PM_2.5_ ​eq	0.00403105
Ozone formation, terrestrial ecosystems	kg NO_x_ ​eq	0.005638093
Terrestrial acidification	kg SO_2_ ​eq	0.010339224
Freshwater eutrophication	kg P eq	0.00067464
Marine eutrophication	kg N eq	6.30E-05
Terrestrial ecotoxicity	kg 1,4-DCB	1.4278474
Freshwater ecotoxicity	kg 1,4-DCB	0.023579723
Marine ecotoxicity	kg 1,4-DCB	0.033241366
Human carcinogenic toxicity	kg 1,4-DCB	0.061105474
Human non-carcinogenic toxicity	kg 1,4-DCB	1.3274998
Land use	m^2^a crop eq	0.16276505
Mineral resource scarcity	kg Cu eq	0.001540514
Fossil resource scarcity	kg oil eq	0.48253294
Water consumption	m^3^	0.012084432

It is worth noting that the use of squid pens as a source of CH provided environmental benefits. Due to the low amount of inorganic compounds and the lack of pigments in squid pens, demineralization and decolorization processes could be eliminated from the CH extraction process and, thus, the use of chemicals and energy consumption associated with those processes were reduced significantly [14]. In addition, the elimination of those processes entailed economic benefits, since the extraction process implied the employment of smaller amounts of resources (materials, energy, and time) and, at the same time, a higher yield of CH was obtained, compared to that of CH extracted from crustacean shells.

**Fig. 1:**
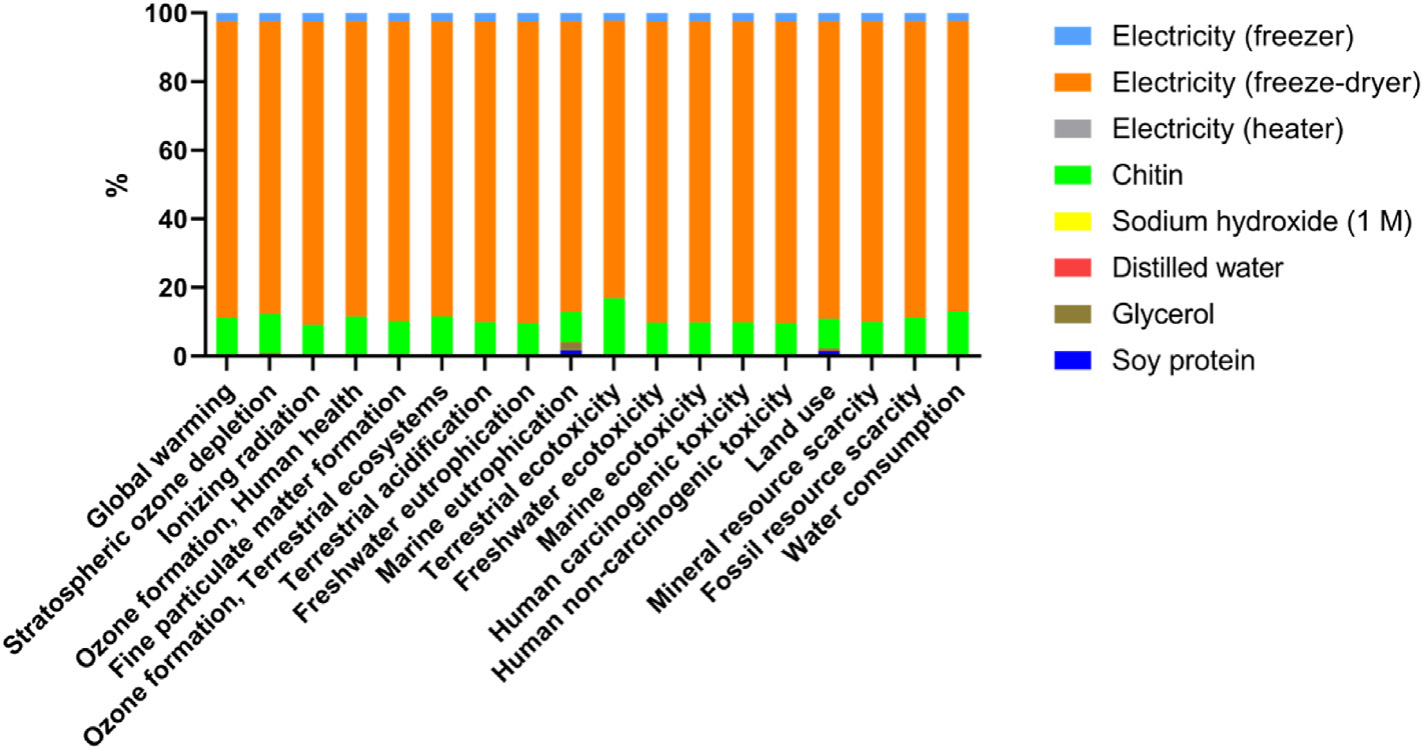
Relative contributions in each impact category for the most relevant processes involved in the entire life cycle of scaffolds. Disaggregating environmental results are displayed in percentage ratios for the most relevant contributing factors.

The relative contributions in each impact category for the most relevant processes involved in the complete life cycle of the scaffolds are shown in Fig. 1. The environmental results obtained are shown in percentage proportions, where it can be seen that the most relevant contributing factor was electricity consumption in extraction and manufacturing processes. We must remember that the environmental impact calculated in this work was based on the results obtained in the laboratory and gave us an idea of which processes should be optimized to be able to reduce the environmental burden, which could be more easily reduced at an industrial scale.

In addition, Fig. 1 shows the environmental results broken down into percentages for each impact category. In this way, the contribution of each stage on the final product could be assessed. In this case, the contributions of the different processes and activities involved throughout the life cycle were calculated as a function of the overall results. As shown in Fig. 1, the stages of obtaining CH required energy and this represented more than 80% of the total impact, becoming the factor with the greatest potential for improvement in decision making when scaling-up. In particular, the energy consumed in the scaffold production, specifically the consumption of electricity, played a critical role in the environmental impact, regardless of the impact category considered. Since the scaffolds were produced at laboratory scale, scaling up the processes would lead to reducing the aforementioned environmental impacts.

### 3.2 Physicochemical characterization

#### 3.2.1 Pore size and porosity

**Fig. 2:**
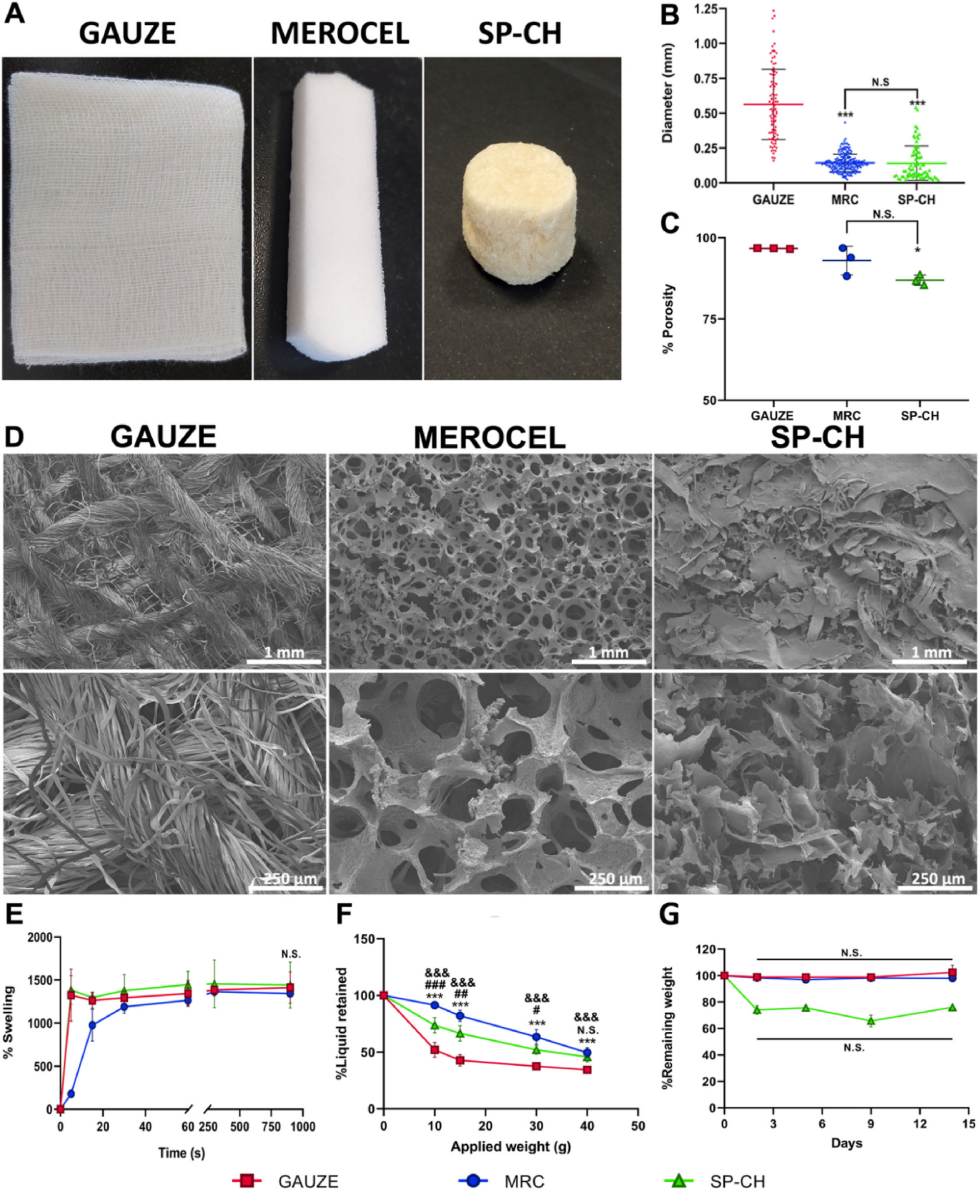
Physicochemical characterization. (A) Macroscopic images of the materials: Gauze, MRC, and SP-CH. (B) Pore size distribution of the materials. (C) Porosity (%) of each material. Error bars, mean ​± ​SD. ∗p ​< ​0.05; ∗∗∗p ​< ​0.001 vs gauze. N.S., nonsignificant (p ​> ​0.05). (D) SEM micrographs of dry materials. (E) Swelling profiles. Error bars, mean ​± ​SD. N.S., nonsignificant (p ​> ​0.05). (F) Liquid retention ratio under different pressures. Error bars, mean ​± ​SD. &&&p ​< ​0.001 gauze vs MRC. #p ​< ​0.05; ##p ​< ​0.01; ###p ​< ​0.001 MRC vs SP-CH. ∗∗∗p ​< ​0.001 gauze vs SP-CH. (G) Weight loss in aqueous medium. Error bars, mean ​± ​SD. N.S., nonsignificant (p ​> ​0.05).

Pore structure of biomaterials that aim to be used as wound healing agents — either to treat hemorrhages or other types of wounds — is of great importance. Differences in the structure of the three materials employed during this study can be appreciated in the macroscopic images (Fig. 2A). Even though, differences in the microestructure are of greater importance. The excellent physicochemical properties of our material were already demonstrated in our previous work [15], but we aimed to compare some of these properties between the three materials. In this vein, the gauze was the material with the highest pore size — 479 ​± ​252 ​μm —, while MRC and SP-CH showed a very similar pore size distribution, 144 ​± ​63 and 141 ​± ​124 ​μm, respectively (Fig. 2B). MRC and SP-CH showed no significant difference in mean pore size (p ​> ​0.05), while both of them showed significant differences with respect to gauze (p ​< ​0.001). Pore size can be closely related to the hemostatic capacity of a material [31]. Ideally, a balance should be sought between large pore sizes that allow RBC and platelet internalization and small pores that provide optimal surface area, required for appropriate cell adhesion [32].

Porosity is required to be high to allow diffusion of nutrients and oxygen [33]. Besides, high porosity is related to elevated surface area, which is of interest for the absorption of wound fluids, either exudates or even blood [34]. As a larger surface area of the material is also related to higher exposition to cells, it can influence cell adhesion and viability as well [35]. In this context, MRC and SP-CH showed similar porosity (Figs. 2C), 92.97 ​± ​4.39% and 86.95 ​± ​1.58%, respectively. Meanwhile, the gauze showed significantly higher porosity than SP-CH, 96.68 ​± ​0.12%. The difficulties associated with a high porosity might be overcome by the weight loss or partial degradation of the nasal pack, which would facilitate the painless removal of the pack. In this sense, it is clear that the SP-CH offer advantages in the removal of the pack compared to MRC and the gauze, even if the three of them present high porosities. On the other hand, SEM micrographs (Fig. 2D) clearly evince that internal structures of the three materials differ. The gauze shows thousands of intertwined fibers forming a mesh, but the space between the fibers does not present well defined pores, which are required for good liquid retention. For their part, MRC and SP-CH show an internal microstructure of well-defined interconnected pores, which in the case of MRC are more circular and in the case of SP-CH present a more amorphous structure. Together with a similar pore size and porosity, this makes MRC and SP-CH very similar regarding to their internal microarchitecture, which completely differs from that of the gauze.

#### 3.2.2 Swelling capacity and liquid retention ration under pressure (LRRP)

The capacity of biomaterials to absorb fluids is important as it will determine their abilities to absorb the blood present in hemorrhages. We tested the water swelling profiles of the three materials by measuring swelling at different time points (Fig. 2E). The three materials showed similar water uptake capacity, with the maximum swelling capacity ranging between 1334 ​± ​34% (MRC) and 1442 ​± ​267% (SP–CH) of its initial weight after 15 ​min. During that period of time, the gauze absorbed 1412 ​± ​182% of its initial weight. Even if the maximum absorption capacity was similar for the three materials, the swelling profile turned out to be quite different. While SP-CH and gauze achieved a 1383 ​± ​168% and 1324 ​± ​302% of their initial weight within only 5 ​s, respectively, MRC could only swell a 178 ​± ​21% of its initial weight during the same time. This shows that even if all of them displayed similar maximum absorption capacity, MRC showed delayed swelling, only equaling SP-CH and gauze after 60 ​s. The water absorption property appears to be especially important in the treatment of epistaxis as the principal way in which nasal packs arrest bleeding is by exerting pressure on the damaged blood vessels of the nasal mucosa to promote hemostasis. After being inserted in the nostril, the nasal pack absorbs wound fluids and expands, maintaining pressure on the wound site. This pressure should be high enough to control the bleeding, but not as high to damage the nasal cavity. Complications associated with excessive pressure include movement of the pack from its initial position [36], obstructed breathing and reduced sense of smell [1], and even necrosis of mucosa and cartilage [25].

Nevertheless, when the pack puts pressure on the nostril's walls, the nostril's walls also put pressure on the pack, thus desorbing part of the previously absorbed fluids. For this reason, we evaluated the capacity of the materials to retain the absorbed liquid under pressure. In this experiment (Fig. 2F) both MRC and SP-CH showed significantly greater (p ​< ​0.001) water retention capacity compared to gauze, independently of the pressure applied. Besides, MRC displayed significantly better retention at low pressures compared to SP-CH, although both materials turned out to similar (p ​> ​0.05) retaining values for pre-absorbed water when the maximum weight — 40 ​g — was applied, with MRC maintaining 49.58 ​± ​4.37% and SP-CH 45.83 ​± ​4.54% of their maximum swelling capacity, while gauze maintained just 34.44 ​± ​2.35%. Therefore, even if the three materials showed similar water swelling capacity, gauze is not able to effectively retain the absorbed water when a pressure is applied, and thus loses its ability to exert pressure to the bleeding site, likely due to its internal microstructure, as commented before. Probably one of the major drawbacks of the pressure exerted by the nasal packs is the discomfort caused to the patient, both with the pack *in situ* and during its removal, with the latter even causing rebleeding, mucosal abrasions, demucosalization and detachment of scars [37]. As pressure is needed to promote hemostasis, these disadvantages always go together with effective nasal packs, as long as they are nonabsorbable.

#### 3.2.3 Weight loss in aqueous medium

Weight loss in aqueous medium is a property of increasing popularity among biomaterials for nasal packings, as it reduces both patient discomfort and rebleeding upon removal, among other advantages [38]. In this vein, both gauze and MRC showed no weight loss during the 14 days of experiment (Fig. 2G), while SP-CH lost 25.96 ​± ​3.22% of its initial weight after 48 ​h. After that measurement, SP-CH showed no more weight loss on the following days (p ​> ​0.05). These results clearly show that both gauze and MRC are totally non-absorbable nasal packs, while SP-CH can lose about a 25% of its initial weight after 48 ​h, due to the glycerol content (30 ​wt %) of the SP-CH dissolving in water. This may probably be enough to relieve patient's discomfort and damage associated with pack removal, and to avoid rebleeding episodes, which would suppose a great advantage both for the patient and the clinician [39]. Besides, it is worth mentioning that in our previous work about the SP-CH [15], we demonstrated that it can be enzymatically degraded within 2 ​h, using collagenase P. Moreover, in this previous work we performed cell-mediated degradation studies in an implanted SP-CH, observing complete degradation *in vivo*. However, since this enzymatic degradation does not represent an epistaxis scenario, we believe that in this case weight loss in aqueous medium and blood mediated degradation are more meaningful.

### 3.3 Mechanical characterization

For appropriate handling and clinical use nasal packs must present good mechanical properties and resistance to deformation, thus alleviating patient's concerns about discomfort during the treatment and providing mechanical stability [40].

**Fig. 3:**
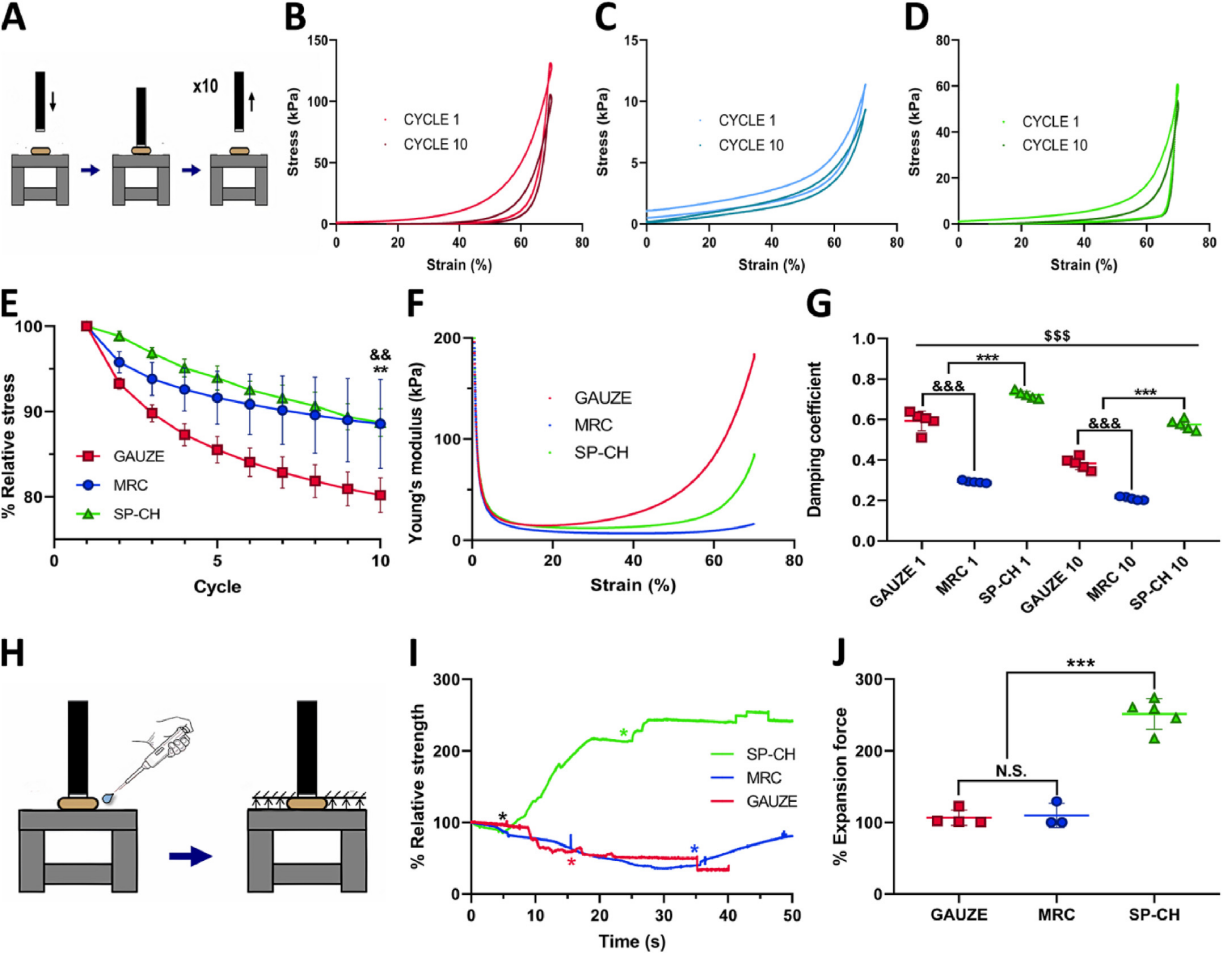
Mechanical characterization. (A) Scheme of the procedure carried out for the cyclic compression test. (B–D) Cyclic compression stress-strain curves of gauze (B), MRC (C) and SP-CH (D). (E) Relative stress (%) of the materials for 10 cyclic compressions. Error bars, mean ​± ​SD. &&p ​< ​0.01 gauze vs MRC. ∗∗p ​< ​0.01 by gauze vs SP-CH. (F) Young's modulus of the materials at different strains. (G) Damping coefficients of the material for cycles 1 and 10. Error bars, mean ​± ​SD. &&&p ​< ​0.001 gauze vs MRC. ∗∗∗p ​< ​0.001 SP-CH vs the other groups. $$$p ​< ​0.001 cycle 1 vs cycle 10 of the same group. (H) Scheme of the procedure carried out for the expansion force test. (I) Relative expansion strength curves of the materials. ∗ indicates the moment at which water was added to each material. (J) Relative expansion force of the materials. Error bars, mean ​± ​SD. ∗∗∗p ​< ​0.001 SP-CH vs the other groups. N.S., nonsignificant (p ​> ​0.05).

We carried out a cyclic compression stress-strain test with 10 cycles (Fig. 3A) to characterize the mechanical properties of the materials (Fig. 3B–D). These curves show an initial plateau region, followed by a hyperelastic region at higher strains, which is more noteworthy for the gauze and SP-CH than for MRC. For the three materials the relationship between stress and strain is nonlinear, exhibiting nonlinear viscoelasticity, similarly to many body tissues and extracellular matrix components [41]. This viscoelasticity could be confirmed by the loading and unloading curves of each cycle taking different paths, which occur due to energy dissipation during deformation of the material [42], producing an hysteresis loop between loading and unloading curves. The area under the loading curve represents the total input energy of the cycle and the area under the unloading curve represents the elastic strain energy, while the area between the two curves accounts for the dissipated energy during the compression cycle [43]. In addition to this, gauze showed the highest stress at a strain of 70%, both for cycle 1 and for cycle 10, while MRC showed the lowest, meaning that gauze was the most difficult material to deform up to a strain of 70%, and MRC the easiest one, which could be due to the internal microstructure of each one. SP-CH required an intermediate stress to be deformed up to a 70% of strain.

Nevertheless, the cyclic compression test revealed that the stress needed to deform the materials decreased substantially cycle after cycle (Fig. 3E), losing their maximum ability to resist deformation. This phenomenon, which is defined as cyclic softening [44], is probably a consequence of irreversible deformations of the internal microarchitecture caused by the compression [45]. Results of the relative maximum stress (%) (Fig. 3E) show that the gauze could only maintain an 80.2 ​± ​2.0% of its maximum stress after 10 cycles, compared to the maximum stress of cycle 1. This is significantly lower (p ​< ​0.01) than MRC and SP-CH, that displayed a relative stress of 87.3 ​± ​5.2% and 88.6 ​± ​1.6% after 10 cycles, respectively.

Young's modulus defines the ability of a material to withstand changes in length when subjected to compressive loads, and it is useful to measure the stiffness of a material [46]: the higher the Young's modulus, the higher the stiffness of the material. Since the three materials present a nonlinear behavior, Young modulus is not constant but changes depending on the position in the stress-strain curve, and is defined by the slope of the tangent to the curve [47]. Thus, we calculated the Young's modulus for all the strains of the first cycle of each biomaterial (Fig. 3F). Initially, the three samples presented a high Young's modulus, which rapidly decreased when strain started to increase. As strained became higher, the Young modulus of gauze and SP-CH started to increase again at strains coinciding with the ones in which the hyperelastic region of each material began — around 25% of strain for the gauze and 50% of strain for SP-CH —. This could indicate that at 25% of strain, all the cotton layers forming the gauze are very compact, and consequently the stiffness of the sample strongly increases. Meanwhile, the internal microstructure of the SP-CH would not get completely compact until a strain of 50%. MRC showed no meaningful increase of its Young's modulus at higher strains, indicating that its stiffness remained low throughout all the compression process. Summing up, while gauze becomes rigid at low strains, both MRC and SP-CH remain flexible until a strain of 50% is achieved.

As commented before, the cyclic loading and unloading produced dissipation of energy within the material, which is usually a result of the viscoelastic behavior of the material or general damage to the structure [48]. Since viscoelastic nature of the three materials could be previously confirmed by the profile of the stress-strain curves and the presence of the hysteresis loop, energy dissipation can be attributed to that viscoelastic behavior. The area of this hysteresis loop — that is, the area between the loading and unloading curves — is directly linked to the damping capacity of the material [49], which is defined by the damping coefficient — or mechanical loss coefficient —, that measures to which extent a material can dissipate vibrational energy [50]. This property depends on factors such as the type of material, internal forces, sizes of geometry and the surface of the material [51], and seems to be of greater importance in biological materials for their protection via damping the impact waves [52]. Damping capacity is commonly high in materials such as foams, elastomers and polymers [50], and therefore they are widely used as damping materials [53]. Since a nasal pack is inevitably subjected to mechanical compression when inserted in the nostril, we evaluated the damping coefficient of the three materials for cycle 1 and 10, calculating it as the ratio of the energy dissipation — area of the hysteresis loop — and the total input energy — area under de loading curve — for the given cycle [51]. For the first cycle, SP-CH presented a damping coefficient of 0.72 ​± ​0.02, which was significantly higher (p ​< ​0.001) than the ones obtained for gauze and MRC, 0.59 ​± ​0.05 and 0.29 ​± ​0.01, respectively. Comparing the first cycle's coefficient to the cycle 10's, all the materials significantly (p ​< ​0.001) lost damping capacity, probably due to irreversible alterations in their internal microstructure caused by the cyclic compression. Even though, SP-CH presented a damping coefficient of 0.57 ​± ​0.03 in cycle 10, which is still significantly higher (p ​< ​0.001) than the ones calculated for gauze and MRC, 0.38 ​± ​0.03 and 0.21 ​± ​0.01, respectively. These results indicate that our material has an increased capacity to dampen the impact waves and mechanical forces that may be produced with the pack placed in the nasal cavity.

To evaluate the upwards force displayed by the materials when they absorb water, we performed an expansion force test, as illustrated in Fig. 3H. This force is representative of the force these materials would display against the nasal cavity's walls when absorbing fluids within the nostril, and therefore this property seems to be of special interest for a nasal pack. Fig. 3I shows the relative expansion strength curves of the materials throughout time, compared to their initial strength. Both gauze and MRC displayed null expansion force when absorbing water, even decreasing their initial strength after adding water to the sample. In contrast, SP-CH's relative expansion curve evinces that when absorbing water, it displays an increasing upwards force, caused by its expansion. This is numerically represented in Fig. 3J, in which the maximum expansion force (%) displayed during the experiment is calculated with respect to the initial expansion force. Results confirmed that gauze and MRC have no capacity to display an upwards force when absorbing water, just increasing the initial expansion force up to a 107 ​± ​11% and 110 ​± ​17%, respectively. Conversely, SP-CH presented an expansion force of 250 ​± ​24% with respect to the start of the experiment, which supposes a significantly higher (p ​< ​0.001) expansion force than for gauze and MRC. Even if high expansion forces can be related to delayed mucosal healing, this is not a common concern in biomaterials with high expansion capacities [54,55]. Thus, we truly believe that a sponge-like material with such a modest expansion force will not cause mucosal damage, but it would probably be able to exert a higher pressure to the bleeding site when absorbing wound fluids within the nostril to promote hemostasis.

### 3.4 Cytotoxicity studies

**Fig. 4:**
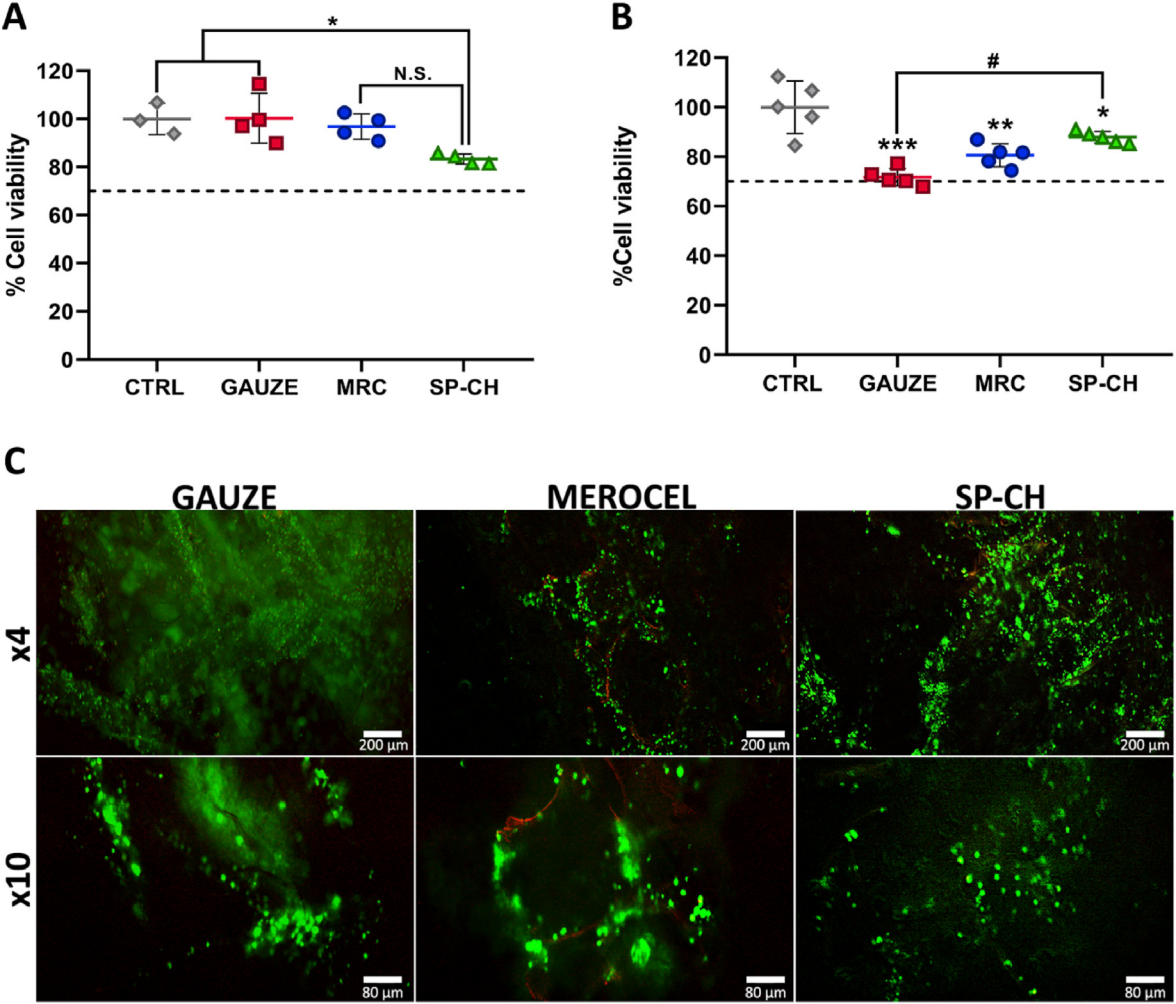
Cytotoxicity studies. (A) Direct cytotoxicity. (B) Indirect cytotoxicity. The line marks the 70% cell viability. Error bars, mean ​± ​SD. N.S., nonsignificant (p ​> ​0.05). ∗p ​< ​0.05; ∗∗p ​< ​0.01; ∗∗∗p ​< ​0.001 vs control group. #p ​< ​0.05 gauze vs SP-CH. (C) Live/Dead micrographs of the cell-cultured materials, with calcein-etidium staining. Scale bars are 100 and 40 ​μm in the above and below rows, respectively.

Direct and indirect cytotoxicity were evaluated in order to confirm the biocompatibility of the materials. A protocol from the ISO 10993 5:2009 guidelines for biological evaluation of biomedical devices was adapted to carry out these assays. Both in direct (Fig. 4A) and indirect (Fig. 4B) cytotoxicity assays the three materials showed cell viability above 70%, and thus their biocompatibility could be confirmed. In both assays, no significant differences were found in biocompatibility between MRC and SP-CH (p ​> ​0.05), but gauze showed significantly higher biocompatibility in comparison to SP-CH in the direct cytotoxicity assay (p ​< ​0.05). In contrast, in the indirect cytotoxicity assay, SP-CH turned out to be significantly more biocompatible than the gauze (p ​< ​0.05).

We also performed the Live/Dead staining method to visually prove that these biomaterials allowed viability of L-929 ​cells cultured upon them (Fig. 4C), with practically all cells alive (green colored) in all the samples, and just a few dead cells — red colored — present in the MRC images. In addition to this, *in vivo* toxicology was already tested in our previous work involving the SP-CH [15], showing adequate biocompatibility. Not only it did not cause an inflammatory response, but it could even inhibit and overcome a LPS-mediated inflammatory response in C57BL/6 mice to a certain extent. All this proves that the SP-CH is adequate for biomedical use without causing any cytotoxicity to the cells present in the nasal cavity and surrounding tissues.

### 3.5 Blood performance

#### 3.5.1 Degradation in blood

**Fig. 5:**
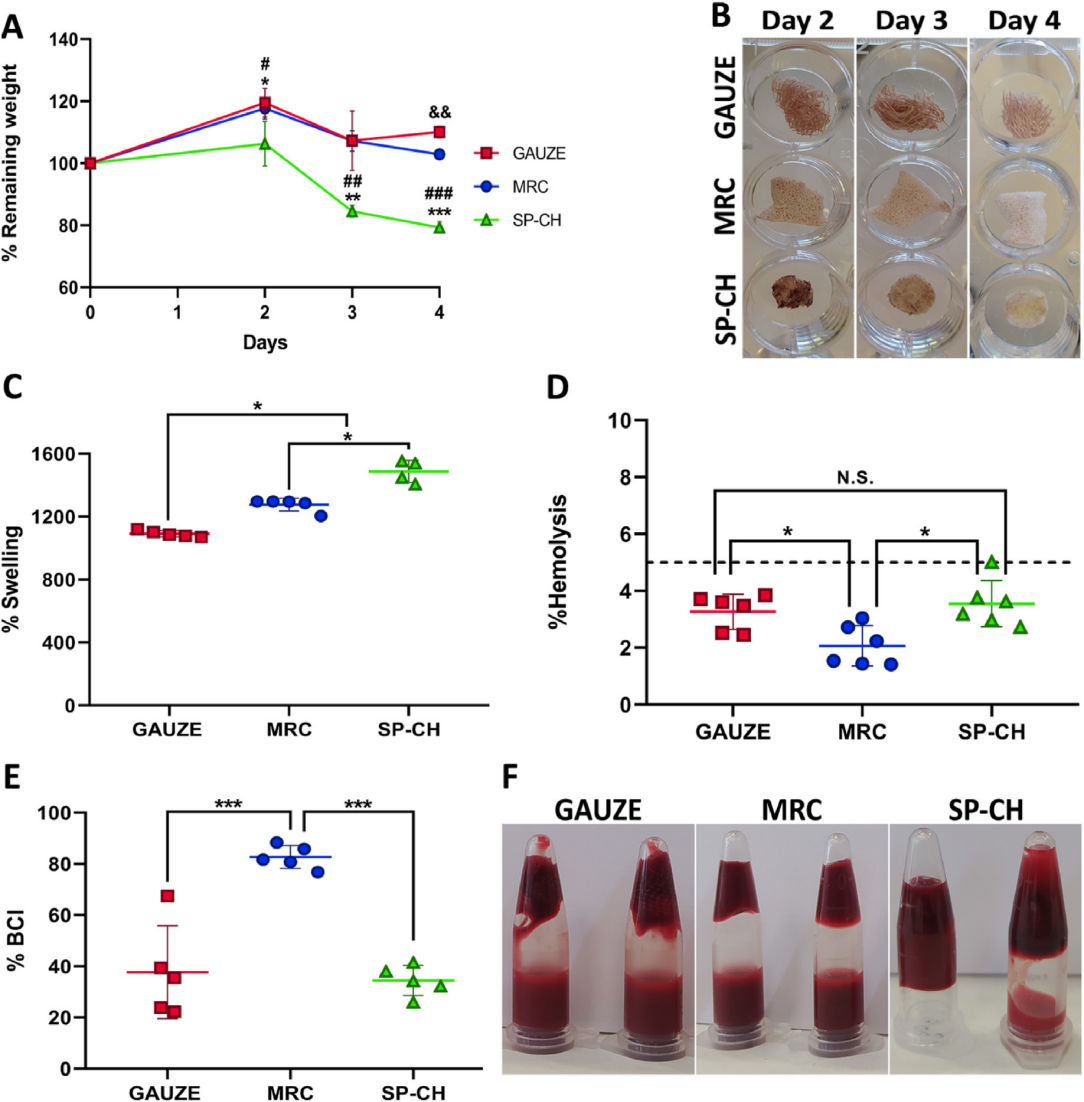
Blood performance. (A) Degradation in blood. Error bars, mean ​± ​SD. &&p ​< ​0.01 gauze vs MRC. #p ​< ​0.05; ##p ​< ​0.01; ###p ​< ​0.001 MRC vs SP-CH groups. ∗p ​< ​0.05; ∗∗p ​< ​0.01; ∗∗∗p ​< ​0.001 gauze vs SP-CH. (B) Images of the plates corresponding to blood degradation after 2, 3, 4 and 7 days. (C) Maximum blood swelling capacity. Error bars, mean ​± ​SD. ∗p ​< ​0.05 between groups. (D) Hemolysis assay. The line marks 5% hemolysis. Error bars, mean ​± ​SD. N.S., nonsignificant (p ​> ​0.05). ∗p ​< ​0.05 between groups. E) Blood Clotting Index of the materials. Error bars, mean ​± ​SD. ∗∗∗ p ​< ​0.001 between groups. (F) Image of the tube inversion method for visually assessing blood coagulation.

To test the degradation profile of the materials in a more biologically relevant fluid, we evaluated their blood degradation profile (Fig. 5A). Results showed that SP-CH degrades in contact with blood, while gauze and MRC do not, as confirmed by the images of the materials after incubation with blood (Fig. 5B). After 4 days of incubation in blood, the gauze presented a weight of 110 ​± ​2% with respect to its initial weight, while MRC presented a remaining weight of 103 ​± ​1%. In contrast, SP-CH maintained a 79 ​± ​2% of its weight after 4 days of incubation in blood, meaning that it could degrade a 21 ​± ​2% of its initial weight during that period of time. It is worth noting that SP-CH maintained its integrity up to the end of the test. This would probably facilitate its removal from the patient's nostril once the nasal pack reaches its goal.

Interestingly, the gauze and MRC not only did not degrade but their weight slightly increased after being incubated with blood — although samples were lyophilized before weighing — to eliminate all the fluids present in the samples. This might be due to blood cells and proteins adhering to these materials, which could not be completely eliminated. This indicates that these materials could have degraded to some level, but that the adherence of these blood components would balance out that degradation. Therefore, the degradation profile of SP-CH could also have been underestimated by the adhesion of blood components as in the case of gauze and MRC, which would mean that our material has even better blood degradation properties. The fact that SP-CH degrades a fifth part of its weight after 4 days, confirms that this material can be used as a partly absorbable nasal pack, with the benefits that this entails, thereby alleviating concerns such as pain during removal and wound healing disruption [56], or decreasing the incidence of nasal adhesions [57].

#### 3.5.2 Blood swelling capacity

Blood swelling capacity is essential for biomaterials intended to be used in hemorrhagic wounds, and seems to be of greater importance than the water swelling profile because of the presence of blood in the site of application. For this reason, we evaluated the maximum blood absorption of the three materials (Fig. 5C). Gauze showed a blood swelling capacity of 1091 ​± ​20% with respect to its initial weight. MRC absorbed significantly more blood than the gauze (p ​< ​0.05), with a mean blood absorption of 1276 ​± ​40%. For its part, SP-CH showed significantly higher blood swelling capacity compared to both gauze and MRC (p ​< ​0.05), absorbing up to 1442 ​± ​117% of blood respect to its initial weight. This increased blood swelling capacity of SP-CH is advantageous from different point of views. On the one hand, higher volumes of blood absorbed would result in a stronger tamponade effect to the injury, which is necessary to arrest bleeding. On the other hand, the more blood volume the pack absorbs the higher amount of RBCs and platelets will be concentrated within the biomaterial, promoting hemostasis and thrombus formation.

#### 3.5.3 Hemolysis assay *in vitro*

We conducted a hemolysis assay (Fig. 5D) in order to evaluate the hemocompatibility of the materials, as biomaterials in contact with blood are required to induce minimal hemolysis — destruction of RBCs in response to shear stress or changes in osmotic pressure [58] —. In this context, the gauze showed a hemolysis rate of 3.27 ​± ​0.62%, while MRC showed significantly decreased hemolysis rate in comparison with both gauze and SP-CH (p ​< ​0.05), exactly 2.1 ​± ​0.7%. SP-CH caused a hemolysis rate of 3.5 ​± ​0.8%, which supposes no significant difference in hemocompatibility with gauze (p ​> ​0.05). Since 5% of hemolysis is the maximum permitted for biomaterials [59], the three tested biomaterials were proved to be non-hemolytic, and the hemocompatibility of SP-CH could be confirmed.

#### 3.5.4 Blood clotting *in vitro*

The ability to induce the clotting of blood is of huge importance for biomaterials intended to treat bleeding wounds such as nasal hemorrhages, and thus we assessed blood clotting *in vitro* for the three materials. For this, we calculated the BCI of the materials (Fig. 5E), with a lower BCI indicating a faster blood-clotting rate [60]. MRC showed the highest BCI, and thus the slowest blood-clotting rate, with an index of 82.7 ​± ​4.5%. The gauze and SP-CH showed significantly lower (p ​< ​0.001) BCI compared to MRC, 37.7 ​± ​18.2% and 34.4 ​± ​5.9% respectively. With the aim of visually assessing the *in vitro* blood clotting capacity of the samples, we performed the tube inversion method (Fig. 5F). This assay reinforced the results obtained in Fig. 5E, confirming the excellent blood clotting capacity of SP-CH, as the blood added to the tubes remained in the upper part forming a strong clot because of the interaction with the SP-CH sponge. For their part, the gauze and MRC were not able to form clots that maintained all the blood in the upper part of the tube, with the majority of the added blood falling to the bottom of the Eppendorf, and thus confirming their decreased hemostatic effect compared to that of SP-CH.

### 3.6 RBC adhesion *in vitro*

**Fig. 6:**
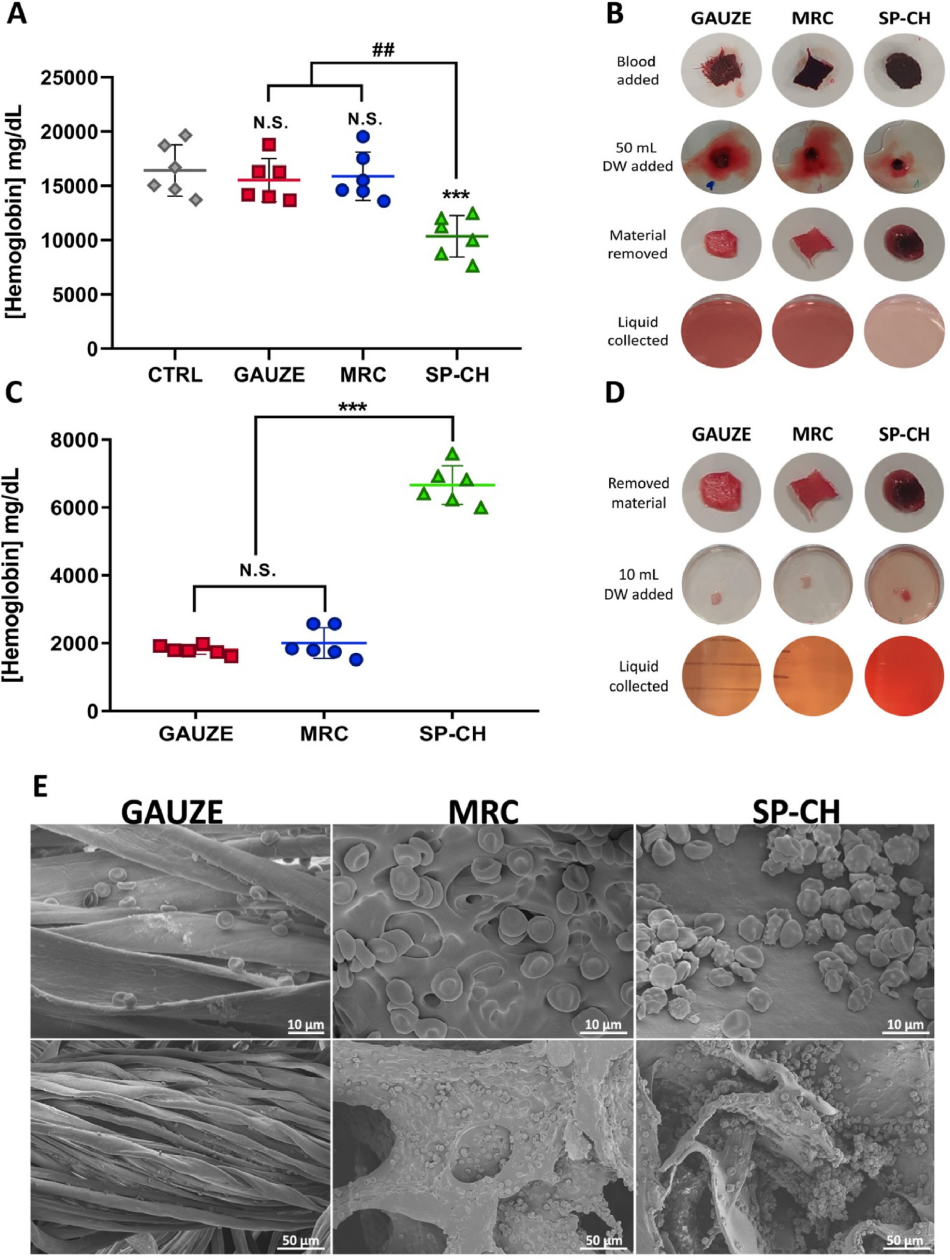
RBC adhesion. (A) Hemoglobin outside the materials. Error bars, mean ​± ​SD. N.S., nonsignificant (p ​> ​0.05). ∗∗∗p ​< ​0.001 vs control group; ##p ​< ​0.01 SP-CH vs the other groups. (B) Photographs of the appearance of the materials at different stages of the determination of hemoglobin outside the materials. (C) Hemoglobin within the materials. Error bars, mean ​± ​SD. N.S., nonsignificant (p ​> ​0.05). ∗∗∗p ​< ​0.001 SP-CH vs the other groups. (D) Photographs of the appearance of the materials at different stages of the determination of hemoglobin within the materials. (E) SEM micrographs of RBCs adhered to the surface of the materials.

Adhesion of RBCs to the surface of the biomaterial is of great importance to promote hemostasis by the formation of an stable and strong blood plug [61]. The measurement of hemoglobin outside the materials (Fig. 6A) represents the amount of RBCs that could not adhere to the material, and thus, a lower hemoglobin concentration outside the material indicates a higher adhesion of RBCs. In this vein, both gauze and MRC showed no significant difference (p ​> ​0.05) compared to a control with no RBC adhesion, showing hemoglobin concentrations of 15,535 ​± ​1977 ​mg/dL and 15,882 ​± ​2231 ​mg/dL, respectively, while in the control group we measured a hemoglobin concentration of 16,245 ​± ​2365 ​mg/dL. SP-CH showed significantly decreased (p ​< ​0.001) hemoglobin concentration compared to the other groups, concretely 10,346 ​± ​1905 ​mg/dL, indicating higher RBC adhesion to the surface of the biomaterial. Fig. 6B shows the appearance of the materials throughout the experiment, and a higher hemoglobin concentration — and consequently a higher adhesion of RBCs to SP-CH — can be visually confirmed.

In addition to this, we assessed the hemoglobin concentration within the materials (Fig. 6C), which can be used as an indicator of the amount of RBCs adhered to the surface of the biomaterials, and thus, in this assay, a higher hemoglobin concentration indicates an increased level of RBC adhesion. Results of this experiment confirmed the outstanding RBC adhesion to SP-CH compared to gauze and MRC. While our biomaterial presented a hemoglobin concentration of 6661 ​± ​571 ​mg/dL, these concentrations were 1801 ​± ​133 ​mg/dL and 2004 ​± ​451 ​mg/dL in the case of the gauze and MRC, respectively. Again, the hemoglobin concentrations — and consequently the RBC adhesion presented graphically — can be visually confirmed in the images of the biomaterials during the experiment (Fig. 6D). The SP-CH sample looked darker due to a higher RBC adhesion, and the finally collected and quantified liquid presented a redder color because of an elevated hemoglobin concentration.

Lastly, we evaluated the RBC adhesion through SEM micrographs (Fig. 6E) of samples of each biomaterial, which had been previously incubated with blood. Even if MRC shows an acceptable amount of RBCs adhered, the surface of SP-CH shows increased concentration of RBCs, forming big aggregates of cells which are adhered both to each other and to the surface of our biomaterial. Conversely, RBC adhesion to gauze is minimal compared to the other materials. In the SP-CH micrographs some morphologically different RBCs can be observed. These abnormal shaped red blood cells are called echinocytes, and the process through which erythrocytes become echinocytes is named echinocytosis. The reasons for echinocytosis to occur are abundant [62,63], including variations of the electrolyte concentrations, a basic pH, intracellular calcium increase, ATP depletion, or the presence of substances such as lysophosphatidic acid (LPA). These stimuli lead to the exposure of phosphatidylserine (PS) on the outer membrane leaflet of red blood cells [64,65], which is related to the blebbing of the plasma membrane that results in echinocytes formation [66]. Even if the implications of echinocytosis and externalization of PS are not clear yet, these events has been described as a signal for RBC apoptosis [64]. However, there are also reasons to believe that the presence of echinocytes can support blood clotting, since their formation leads to the development and release of microvesicles [67,68]. These RBC-derived microvesicles have been said to have a procoagulant activity and to promote thrombin generation [[69], [70], [71]], while hindering anticoagulation processes [70]. This might be due to the exposed PS providing a site for assembly to the prothrombinase and to the tenase enzymes [71]. The presence of echinocytes has been already described in other studies involving hemostatic materials, adhered to the surface of hemostatic agents [72] and even of standard gauzes [73], without supposing any negative effect. However, the real function and mechanisms involving echinocytes are not fully understood yet and need further investigation.

These results elucidate the hypothetical hemostatic mechanism of SP-CH, as it has a great capacity to absorb blood and bind the RBCs, concentrating them at its surface. In this sense, the role of RBCs in hemostasis is gaining interest, and recent studies indicate that RBCs migrate to the bleeding site and push platelets toward the endothelium — in a process called margination — in a hematocrit- and shear-dependent manner. They also increase blood viscosity, and thus resistance to blood flow [74], which in turn would further promote RBC aggregation, leading to a vicious cycle that would provoke reduced tissue perfusion [75]. In addition, RBCs can activate platelets and promote their aggregation [76].

### 3.7 Platelet adhesion *in vitro*

Undoubtedly, platelets are fundamental in the initial stages of hemostasis, being their activation required for the activation of several coagulation factors [77]. When a vessel wall is injured, platelets interact with vascular cells, ECM components and the coagulation system [78]. At this point, platelet adhesion to the damaged endothelium increases, which unleashes a signaling cascade — through tyrosine kinases and G-protein coupled receptors — that will provoke additional recruitment and activation of more platelets. This finally leads to the presentation of a procoagulant surface that promotes the creation of a fibrin-rich hemostatic plug at the bleeding site [79]. There, platelets show an activation gradient, from a dense core region of the hemostatic plug where the platelets are highly activated and expose *P*-selection, to an outer loosely packed region with decreased platelet activation and absent *P*-selection exposure [78]. During platelet recruitment to the injury site, platelets become activated through diverse receptor-specific signaling pathways. Once activated, platelets start to secrete granules — such as δ-granules, which contain ADP, serotonin, polyphosphates, glutamate, histamine and calcium — and undergo shape change, being both vital for the hemostatic process [80].

**Fig. 7:**
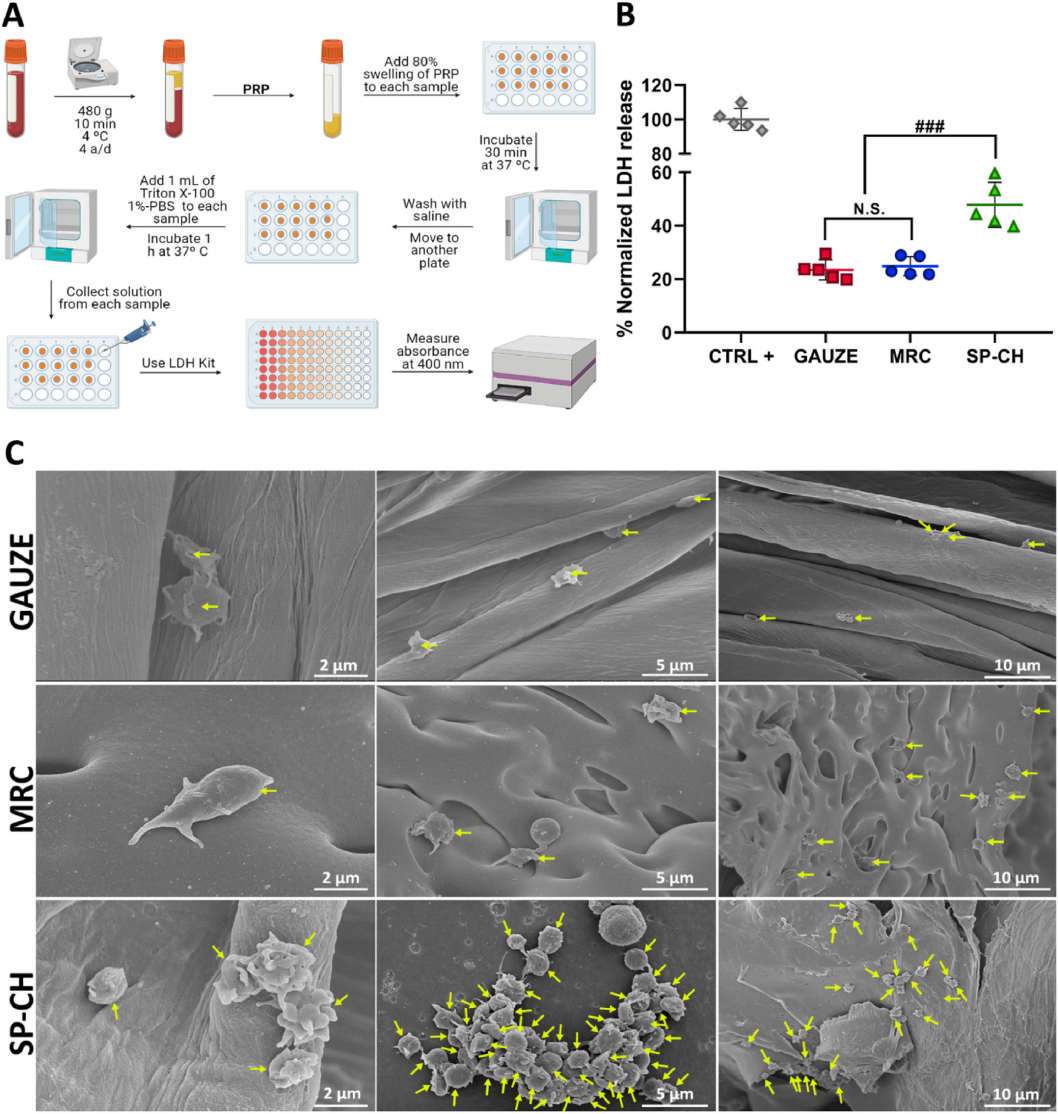
Platelet adhesion. (A) Scheme of the procedure carried out for the determination of platelet adhesion to the materials. (B) Normalized LDH release (%) of the materials. Error bars, mean ​± ​SD. N.S., nonsignificant (p ​> ​0.05). ###p ​< ​0.001 SP-CH vs the other groups. n ​= ​5 independent samples per group. (C) SEM micrographs of platelets adhered to the surface of the materials. Platelets attached to the materials are marked with yellow arrows.

For this reason, any biomaterial intended to be used with hemostatic purposes should show a good platelet adhesion capacity to its surface to concentrate platelets in the wound site and promote hemostasis. We evaluated the platelet adhesion of the three materials using an indirect method (Fig. 7B) that measures the amount of LDH released from a sample, which is directly proportional to the amount of platelets within it. Both gauze and MRC showed decreased platelet adhesion (Fig. 7A), with a LDH release of just a 23.46 ​± ​3.75% and 24.86 ​± ​3.61%, respectively, of the one measured for a positive control in which all the added platelets were lysed. For its part, SP-CH presented a LDH release of a 47.8 ​± ​8.4% of that measured for the positive control, which means a significantly higher (p ​< ​0.001) platelet adhesion compared to both the gauze and MRC. SEM micrographs of the materials cultured with PRP (Fig. 7C) confirmed the quantitative results obtained in the *in vitro* platelet adhesion assay, showing that platelet adhesion to gauze and MRC is minimal, while SP-CH shows bigger amounts of platelets adhered to its surface, forming aggregates of platelets, with even some of them activated.

When platelets are forming activated aggregates, they generate a phosphatidylserine-exposing membrane surface, which is essential for the formation of thrombin [78]. In turn, thrombin activates platelets — through hydrolyzation of G-protein- coupled protease-activated receptors (PAR) 1 and 4 [81] — and promotes clot stabilization by converting of fibrinogen to fibrin [82].

We hypothesize that this platelet-thrombin interaction, that boosts the formation of the hemostatic plug, can be influenced somehow by the SP-CH. This is because SPI is a source of phylloquinone — vitamin K1 — [83], which certainly is involved in blood coagulation. Vitamin K serves as a cofactor for the endoplasmic enzyme g-glutamate carboxylase (GGCX), which plays a vital role in the carboxylation of certain protein-bound glutamate residues into g-carboxyglutamate (Gla) [84]. Seven blood coagulation factors — including prothrombin, fVII, fIX, and fX [85] — require this K vitamin-dependent glutamate residue carboxylation in order to bind Ca^+2^. Therefore, this carboxylation is essential for the formation of ion bridges between the blood-clotting enzymes and phospholipids on platelets’ membranes [86], promoting the activation of these coagulation factors.

In addition, it has also been mentioned that the SPI present in the SP-CH has RGD-motif containing peptides [16], which could explain its ability to bind platelets. This is because the RGD sequence is also present in the α-chains of fibrinogen [87], and it is one of the two motifs that bind to the αIIbβ3 integrin receptor of platelets to boost their aggregation [88]. The interaction of these RGD sequences with platelet integrin αIIbβ3 has been recently described using optical trap-based methods, and the importance of this motif in the hemostatic process has been highlighted [89]. In fact, RGD sequences are recently being used to target platelets with different objectives [90]. Thus, we hypothesize that the increased platelet adhesion capacity of our SP-CH is due to the interaction between the RGD-motifs of the SPI and the αIIbβ3 integrin receptor of the platelets.

### 3.8 *IN VIVO* hemostatic efficacy

**Fig. 8:**
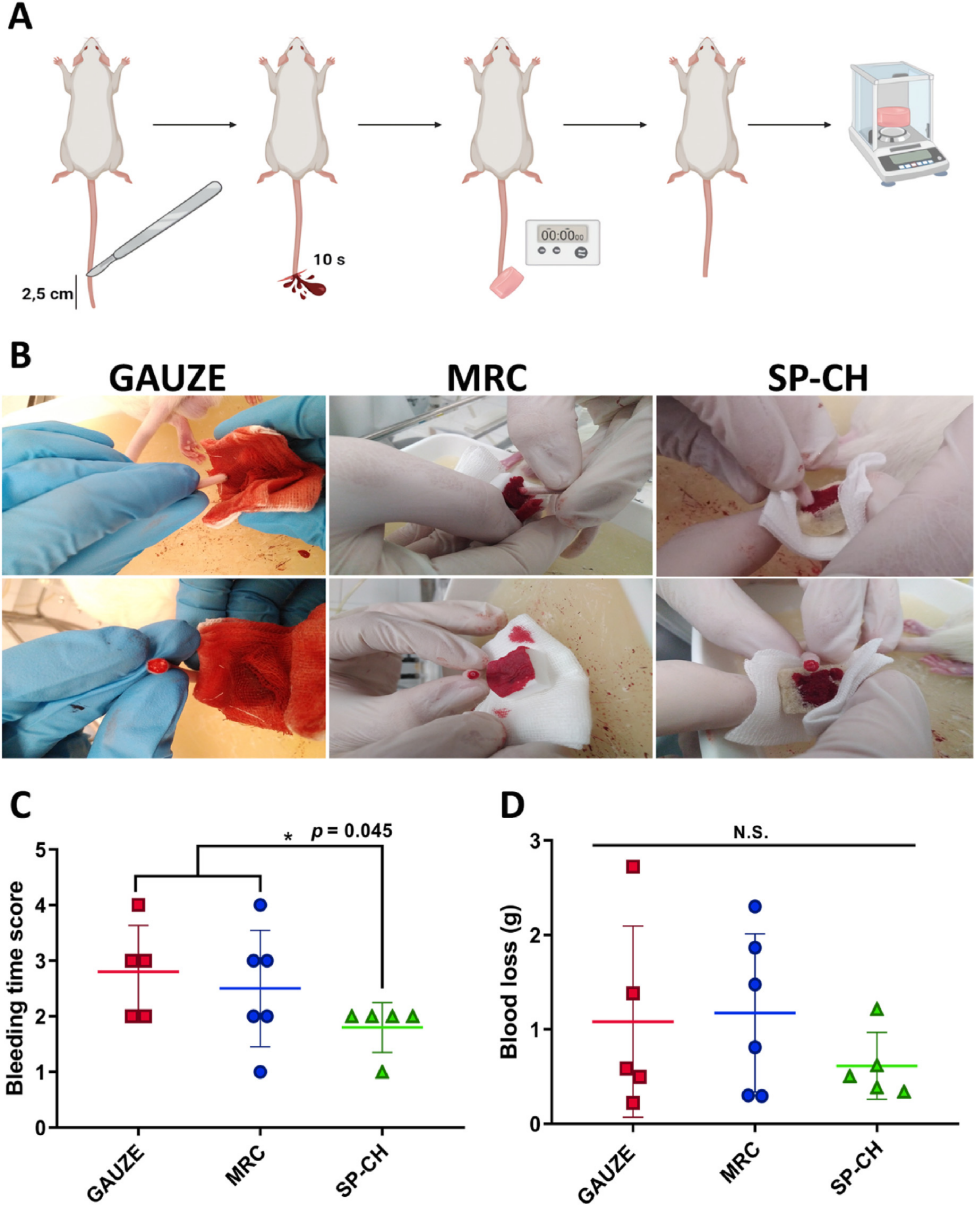
*In vivo* hemostatic efficacy. (A) Scheme of the procedure carried out for the rat-tail amputation model. (B) Photographs of the three materials during the experiment. (C) Bleeding time score of the three materials. Error bars, mean ​± ​SD. ∗p ​< ​0.05 SP-CH vs the other groups. (D) Blood loss (g). Error bars, mean ​± ​SD. N.S., nonsignificant (p ​> ​0.05).

A rat-tail amputation model (Fig. 8A) was used to evaluate the *in vivo* hemostatic properties of the three materials. Photographs of the appearance of the materials (Fig. 8B) were taken during the experiment. The gauze obtained a mean bleeding time score of 2.8 ​± ​0.8 (Fig. 8C), which was similar to MRC's, 2.5 ​± ​1.0. Meanwhile, SP-CH obtained a significantly lower (p ​> ​0.05) bleeding time score, 1.8 ​± ​0.4, meaning that it could promote hemostasis *in vivo* faster than both gauze and MRC. Regarding to blood loss (Fig. 8D), no significant differences were found between groups. The gauze presented a mean blood loss of 1.1 ​± ​1.0 ​g, MRC 1.2 ​± ​0.8 ​g and SP-CH 0.6 ​± ​0.3 ​g. However, it is noteworthy that both gauze and MRC showed an increased deviation among replicates, while blood loss in the SP-CH group remained consistent in all the tested samples, guaranteeing a homogeneous hemostatic efficacy.

We consider that a lack of a suitable epistaxis animal model supposes a limitation in our study. Indeed, even if *in vivo* epistaxis models exist [91,92] they either involve large size animals or fail to faithfully reproduce a real case of epistaxis, since an external hemorrhage is induced and the treatment is not introduced in the nasal cavity. Therefore, for the initial evaluation of the general hemostatic properties of our SP-CH, we aimed to choose a model with an adequate relationship between relevance and reduced invasiveness. In this vein, the rat-tail amputation model has been the animal model of choice for many important studies involving the evaluation of hemostatic properties of biomaterials [[93], [94], [95], [96]], and hence it seems to be an appropriate model for this objective. Undoubtedly, a bigger sample size would have allowed detecting bigger differences between the assayed groups. We understand that the experiment might be underpowered, but it is still totally valid, and viable from an ethical point of view, respecting the principles of the 3R. In this context, the bleeding time results support those obtained in the *in vitro* experiments, where SP-10.13039/100003698CH showed superior hemostatic properties compared to the gauze and MRC.

## 4 Conclusions

In this study we demonstrated that by-products of the food industry are a valuable and sustainable source of biomaterials that can be employed to manufacture safe and effective nasal packs with great hemostatic properties. The developed SP-CH presents a greatly interconnected porous microstructure, great water and blood swelling capacity and appropriate retention of the absorbed fluids. Besides, mechanical properties of the biomaterial demonstrated to be adequate for its use as a nasal pack. The biomaterial presented excellent biocompatibility and hemocompatibility *in vitro*. Interestingly, it was able to lose weight in aqueous medium and to degrade partially within a few days when incubated in blood, which is becoming more and more important characteristic for nasal packs. Moreover, the hemostatic properties of our SP-CH outperformed those of the two nasal packs used in the clinical routine worldwide: a standard gauze and the commercial synthetic pack Merocel®. Our biomaterial was found to effectively promote blood coagulation *in vitro*, showing outstanding RBC and platelet binding properties compared to the gauze and MRC, likely due to the intrinsic hemostatic properties of its natural components. Although further research of its hemostatic effect *in vivo* is needed, SP-CH significantly shortened bleeding time in a rat-tail amputation model. Additionally, the study of the environmental loads associated with the extraction of materials and the manufacturing of scaffolds showed low environmental impacts in all the categories analyzed. All in all, our SP-CH, produced from a renewable and sustainable source of biomaterials (by-products of the food industry), showed superior mechanical and hemostatic properties compared to Merocel® and a standard gauze. Thus, we have demonstrated that a green and ecofriendly strategy can be followed to develop a biomaterial that outperforms the gold standard in the treatment of epistaxis. This leads us to believe that our SP-CH may be an appropriate nasal pack candidate for the treatment of epistaxis.

## CRediT authorship contribution statement

**Jon Jimenez-Martin:** Methodology, Investigation, Writing – original draft. **Kevin Las Heras:** Methodology, Investigation, Writing – original draft. **Alaitz Etxabide:** Investigation. **Jone Uranga:** Investigation. **Koro de la Caba:** Resources, Funding acquisition, Writing – review & editing. **Pedro Guerrero:** Investigation, Writing – review & editing. **Manoli Igartua:** Resources, Funding acquisition, Writing – review & editing. **Edorta Santos-Vizcaino:** Conceptualization, Methodology, Resources, Supervision, Funding acquisition, Writing – review & editing. **Rosa Maria Hernandez:** Conceptualization, Methodology, Resources, Supervision, Funding acquisition, Writing – review & editing, Project administration.

## Declaration of competing interest

The authors declare that they have no known competing financial interests or personal relationships that could have appeared to influence the work reported in this paper.
